# Why Are Women Law Enforcement Officers More Burned-Out and What Might Help Them?

**DOI:** 10.4172/2329-6879.1000204

**Published:** 2015-06-11

**Authors:** Diane Elliot, Bharti Garg, Kerry Kuehl, Carol DeFrancesco, Andriana Sleigh

**Affiliations:** Division of Health Promotion & Sports Medicine, Oregon Health & Science University, Oregon, USA

## Introduction

Women police officers are an asset to law enforcement organizations. Compared to their male counterparts, they rely more on communication skills to manage interactions, and they are less likely to use physical force. In addition, the single largest police call category nationwide is violence against women, and women officers are more likely to effectively respond to those calls [1]. Despite those findings, women are a minority of law enforcement officers (LEOs), and efforts to increase their recruitment and retention have had limited success [2]. Burnout may lead to lower job performance and leaving an occupation or work setting [3–5]. Understanding burnout and its correlates might allow developing programs to enhance and extend women LEOs’ careers.

This report is a sub-study of a randomized controlled trial of a worksite wellness and safety program for LEOs [6], and in addition to psychological dimensions, information was gathered concerning diet, exercise, stress, sleep and fatigue. Although studies of the link between burnout and a healthy lifestyle are limited, cross-sectional investigations appear to indicate a reciprocal relationship. Those manifesting burnout were more likely to report low physical activity and greater obesity [7]. Conversely appropriate sleep patterns, regular physical activity and a healthy diet may attenuate feelings of burnout [8]. We assessed differences between women and men LEOs concerning burnout, demographics and lifestyle habits and compared women LEOs stratified on the burnout dimension to identify relationships that might inform subsequent efforts to prevent and remediate burnout among women LEOs.

## METHODS

### Participants

The SHIELD (Safety & Health Improvement: Enhancing Law Enforcement Departments) study is a randomized controlled trial of a health and safety intervention for law enforcement personnel. One police department and two sheriff offices from Oregon and southwest Washington were recruited for participation in this study. The police department employed approximately 200 sworn staff, and there were a total of 490 sworn employees in the two sheriff offices. Four hundred and eleven individuals consented to participate, and excluding administrative staff, 309 LEOs participated in the initial assessment. All participants completed informed consent, and the study procedures were approved by the Institutional Review Board of Oregon Health & Science University.

### Assessment

All study participants completed a written survey and physical measurements, including seated blood pressure, height, and weight. The instrument assessed demographics (e.g., race/ethnicity, marital status, education, years of service), and items using constructs with established reliability from our previous studies and published instruments. Most items used a seven-point agreement scale, ranging from strongly disagree to strongly agree. Higher scores on the burnout, stress and fatigue/sleep deficit constructs are unhealthier. Construct reliability and scale components are shown in Table 1.

Burnout was assessed with questions from the emotional exhaustion subscale of the Maslach Burnout Inventory (MBI) [9, 10], which is a 16 item survey with dimensions of emotional exhaustion, cynicism and professional efficacy. The MBI is the most common index of burnout [10], and the emotional exhaustion subscale is the most widely reported and analyzed component [11].

Lifestyle dimensions were assessed using previously validated instruments (MacKinnon et al., 2010). Daily servings of fruits and vegetables were indexed using the NCI screening instrument [13]. Sleep duration/quality and fatigue were assessed using items from the Pittsburgh Sleep Quality Index and the sleepiness scale [14, 15]. Hours of sleep were the self-reported estimate of hours per day over the last month.

### Statistical analyses

Genders were compared by t-test and chi-square tests for continuous and categorical values, respectively. Construct reliability was assessed by calculating Cronbach’s alpha. Women LEOs were stratified by the burnout emotional exhaustion construct, and the highest and lowest tertiles compared by t-test and chi-square tests. Multiple linear regression was used to assess the relationship of burnout with gender after adjusting for confounders. Because of the multiple comparisons assessed, significance was set at the *p*<0.01 level.

## Results

### Comparison of Women and Men LEOs

Three-hundred and nine LEOs (67 females and 242 males) participated, and descriptive statistics for both groups are presented in Table 2. Women and men LEOs were similar in age (mean [SD]) (42 [9] yrs) and years on the force (15 [8] yrs). A higher percentage of male LEOs were married (*p*<0.001), although the percentage of married female LEOs still was higher than the national average [16].

Overall, women were significantly more burned-out than men (*p*<0.0001). Concerning lifestyle habits, female LEOs had increased burnout despite healthier eating habits, with greater fruit and vegetable intake (*p*<0.001), and their body mass indices was not differ from male LEOs. Blood pressure was significantly lower among women LEOs. However, that finding is consistent with other cross-sectional comparisons of women’s and men’s blood pressures [17].

Interestingly, stress among peers was higher for women (*p*<0.0001), with a trend toward greater personal stress (*p*=0.02). Also women experienced greater fatigue/sleep deficit (*p*<0.01). Among both women and men LEOs, burnout correlated significantly with stress, depression and fatigue/sleep deficit (*p*<0.0005, for each dimension for both sexes). No significant correlations were observed for burnout and other measures for either women or men LEOs. After adjusting for marital status and children at home, women continued to have a higher odds ratio of being burned out than men (95% Confidence Interval: 0.42–1.16, *p*<0.001).

### High versus Low Burnout Women

When women LEOs were stratified based on their burnout levels, the bottom and top tertiles were similar in age, years in service, work schedule, marital status, and percentage with children at home (Table 3). Women LEOs with higher burnout scores had greater feelings of depression, personal stress and perceived stress among their peers (*p*<0.001 for each). They also reported greater fatigue/sleep deficit (*p*<0.005).

## Discussion

Women LEOs have higher burnout scores than their male colleagues, a finding not related to age, years of service, marital status or having children at home. Greater burnout was observed despite women having healthier eating habits and similar physical activity levels. When compared to their less burned-out female coworkers, higher burnout women LEOs also experienced greater feelings of depression and perceived that they and their peers were more stressed.

In general, gender has not been a strong predictor of burnout, and no consistent gender differences have been observed [18]. Burnout has received little study among LEOs in recent years. However, their stress levels have been assessed, and as we also found, it has been higher among women officers [19]. Potential explanations for greater personal stress and perceived stress among peers include greater sexual harassment among women, lack of acceptance and mentoring by the predominantly male profession and higher overall workloads due to unequal division of household tasks [20–22].

Stress and feelings of depression are not synonymous with burnout, as burnout is a work or situation specific construct, while depression relates to every life domain [11]. Studies among athletes have indicated that while stress can be a precursor to burnout, other dimensions such as optimism can influence whether stress leads to burnout [23]. Those presumed relationships relate to the rationale for improving coping as a means to prevent burnout [24]. Traditionally, job stress has been approached through worksite health promotion, such as programs advocating regular exercise and healthy eating habits [25]. However, we found that women LEOs with burnout already had healthy diets, and physical activity levels did not correlate with burnout. Sleep deficit was the only lifestyle aspect that related to burnout, a finding recently recognized in other settings [26–29]. A potential vicious cycle may occur, as disturbed sleep patterns can be the result of stressful work [30–32], with sleep deficiency decreasing ability to cope with stressful events [33], augmenting their impact and leading to burnout.

Our findings are cross-sectional and cannot establish causality. They represent only one geographic area, and although the overall participation rate was relatively high for worksite programs, potential enrollment biases also may limit generalizability. However, the results highlight the high prevalence of burnout among women LEOs and the limited potential to impact burnout through the typical worksite wellness program recommendations of eat healthy and get regular exercise. The higher fatigue and sleep deficit identified do offer a potential mitigation means. The greater risk of cardiovascular disease among women LEOs has been attributed to stress [19], but sleep deficit may be a more immediately reversible factor contributing to cardiovascular disease [33]. Emphasizing the importance of adequate sleep may be a means to improve the performance, health and work retention of women LEOs.

## Figures and Tables

**Table 1:** Construct Items and Reliability

	Reliability (α)
**Burnout**	0.71
I feel emotionally drained from my work I feel I am working too hard at my job I feel used up at the end of the workday	
**Depression**	0.84
Overall my job makes me feel depressed	
**Stress self**	0.80
I feel significant stress at work Worrying about work issues makes it hard to relax at home Overall I feel there is too much stress in my job In general I manage stress in a healthy way*	
**Stress peers**	0.75
Overall the stress level among my coworkers is high Overall the unhealthy stress level among members of our department is high My coworkers feel significant stress at work Currently my department is stretched too thin and often too busy My coworkers are able to manage their stress*	
**Sleep deficit/fatigue**	0.79
In the past 7 days I felt satisfied with my sleep* In the past week my sleep quality was good* I usually don’t get enough time between work shifts to recover my energy fully. I have plenty of reserve energy when I need it* In the past 7 days I have had a hard time getting things done because I was sleepy	

* Reverse coded

**Table 2:** Comparison of Women and Men Law Enforcement Officers (mean [SD])

	Males (n=242)	Females (n=67)	*p*
Age	41.6 (8.5)	43.2 (10.0)	0.19
Years of service	15.0 (8.4)	13.1 (7.8)	0.11
Married (%)	81.5%	60.6%	<0.001
Percent children at home	70.3 %	53.2%	0.01
Estimated hours sleep/24 hours	6.4 (1.0)	6.2 (1.5)	0.07
Burnout	3.8 (1.3)	4.6 (1.3)	<0.0001
Depression	2.7 (1.2)	2.6 (1.1)	0.42
Stress self	3.8 (1.3)	4.2 (1.3)	0.02
Healthy eating knowledge	5.7 (0.8)	5.8 (1.0)	0.65
Servings of fruits and vegetables/day	5.3 (3.9)	7.5 (5.8)	<0.001
Physical activity knowledge	6.3 (0.7)	6.3 (1.0)	0.74
Physical activity self	2.4 (1.5)	2.5 (1.6)	0.93
Sleep deficit/fatigue	3.5 (1.1)	3.9 (1.3)	<0.01
BMI	29.7 (4.7)	28.6 (5.6)	0.08
BP systolic	128.2 (12.6)	120.1 (12.6)	<0.001

**Table 3:** Low and High Burned-out Women LEOs (mean [SD])

	Lower Burned-out (n = 23)	Higher Burned-out (n = 23)	*p*
Age	42.4 (9.9)	43.2 (10.5)	0.82
Years of service	12.3 (8.4)	12.3 (6.3)	1.00
Married	65.2 %	69.5%	0.82
Percent children at home	45.4%	54.5%	0.66
Estimated hours sleep/24 hours	6.5 (1.3)	5.9 (1.5)	0.29
Burnout	3.3 (0.6)	5.9 (0.6)	<0.001
Depression	1.9 (0.8)	3.3 (1.2)	<0.001
Stress self	3.3 (1.0)	5.1 (1.1)	<.0001
Stress peers	4.6 (0.7)	5.8 (0.8)	<.0001
Healthy eating knowledge	6.0 (0.7)	5.9 (1.1)	0.69
Healthy eating self	4.8 (1.3)	4.2 (1.5)	0.15
Servings of fruits and vegetables / day	6.4 (4.3)	7.5 (3.9)	0.33
Physical activity knowledge	6.4 (0.7)	6.3 (1.3)	0.87
Physical activity self	2.5 (1.4)	2.5 (1.7)	0.95
Sleep deficit/fatigue	3.3 (0.9)	4.4 (1.1)	<0.005
BMI	27.4 (3.8)	29.9 (7.1)	0.13
BP systolic	119.7 (14.6)	120.9 (10.9)	0.74
BP diastolic	74.0 (9.7)	76.7 (9.0)	0.32
